# Enjoyment of spinning exercise performed in a group session as compared to an individual session

**DOI:** 10.3389/fspor.2024.1501862

**Published:** 2025-01-31

**Authors:** Katrine Trøstrup Beck, Karoline Sofie Lowater, Jan Rafn, Ernst Albin Hansen

**Affiliations:** Centre for Health and Rehabilitation, University College Absalon, Slagelse, Denmark

**Keywords:** exercise, indoor cycling, endurance training, fitness, physical activity, spinning, training

## Abstract

**Introduction:**

The degree of perceived enjoyment of performed physical activity may be a key aspect with relevance for the effort to get people to be physically active. Spinning, or indoor cycling, is a widespread physical activity that can be performed in a group or individually. The effect of the group element on the enjoyment of spinning remains unclear. Therefore, the purpose of the present study was to test the hypothesis that enjoyment was greater during spinning exercise performed in a group session as compared to individually.

**Methods:**

Twenty recreationally active persons (56 ± 6 years, 1.74 ± 0.09 m, 81.0 ± 14.7 kg, and BMI of 26.5 ± 3.7) performed 44-min group and individual instructor-led spinning sessions. Values of power output, heart rate, and cadence were recorded during the sessions. Perceived enjoyment of the exercise was determined by means of a questionnaire (PACES-8) after the exercise.

**Results:**

Values of power output, heart rate, and cadence were not different between the two sessions. Enjoyment was statistically significantly greater by 1.4 ± 2.1 points (*p* = 0.005) during spinning performed in a group session as compared to individually. As a reference framework, 56 points is the maximal sum score.

**Conclusions:**

The difference in enjoyment between conditions was modest and clinically insignificant. In other words, the group element of the spinning session was considered to be of minor importance for the participants' perception of enjoyment.

## Introduction

Physical activity and exercise are activities that play a key role in order to maintain and increase public health (1). However, long-term maintenance of physical activity and exercise is difficult to achieve for many persons. In order to support these persons, we need to be able to provide evidence-based exercise recommendations rather than recommendations based on myths, anecdotes, and traditions.

Enjoyment of recently performed physical activity or exercise may be a key aspect with relevance for long-term maintenance of being physically active (2). This underscores the importance of studying enjoyment in exercise. In connection with previous research it has been reported that “…when groupness was higher during fitness classes, greater exertion was reported and exercisers held higher perceptions of enjoyment and affective valence” (3).

Spinning, or indoor stationary cycling, is a specific type of exercise, which is widespread and popular. Spinning is often executed in group sessions, performed in training centers. However, it can also be performed individually, for example at home with a screen-based instruction.

The combined effect of having an instructor and at the same time performing cycling in a group, as compared to performing cycling alone, has been investigated previously in 18 female university students. It was found that participants enjoyed the instructor-led group session more than the self-regulated individual session (4). However, because of the combined intervention, the study (4) could not reveal whether the group element, in itself, is favorable for enjoyment. And that served as inspiration for the present study.

The independent impact of the group setting on spinning enjoyment remains unexplored. Therefore, the purpose of the present study was to test the hypothesis that enjoyment was greater during spinning exercise performed in a group session as compared to individually.

## Materials and methods

### Study design

The study was designed as a crossover trial. Two spinning sessions were performed by each participant, with 14 days of separation. It was assessed that two weeks was sufficient time to prevent notable influence of one session to the other session. To control for order effects, the sequence of the group and individual sessions was counterbalanced. Participants were randomly assigned to start with either the group or individual session. This randomization and counterbalancing aimed to mitigate potential biases due to the order of session types. The only difference between the two spinning sessions was that in the group session, about 20 persons performed the spinning program together in a training room. In the individual session, the participant was alone with the instructor.

### Participants

Twenty recreationally active persons participated. The participants were recruited from a local training center in which the persons regularly participated in group spinning classes. Written informed consent was obtained from all participants. The participants were blinded to the study's hypothesis to minimize bias. The study conformed to the standards set by the Declaration of Helsinki as well as by the procedures stated by The Danish National Center for Ethics. In Denmark, quality assurance studies do not require approval by a Research Ethics Committee.

### Participant characteristics

For characteristics of the participants, the reader is referred to Table 1. The maximal heart rate was estimated for each participant, according to the following equation 1, developed previously (5):

(1) Maximalheartrate(inbeatspermin)=208–(0.7×ageinyears)

**Table 1:** Characteristics of the participants. *n* = 20. Data are presented as mean ± SD.

Age (years)	Height (m)	Body mass (kg)	BMI (kg m^−2^)	Maximal heart rate^a^ (beats min^−1^)	FTP^b^ (W)	Sex (F/M)
56 ± 6	1.74 ± 0.09	81.0 ± 14.7	26.5 ± 3.7	169 ± 4	195 ± 43	11/9

^a^ Estimated based on age, according to Tanaka et al. (5). FTP, functional threshold power output.

^b^ Self-reported by the participants. F, female. M, male.

Functional threshold power output, FTP, was self-reported by each participant and used for determination of the target exercise intensity and visual feedback during cycling. The functional threshold power resembles the average power output, which can be sustained for 60 min cycling. BMI was calculated as body mass (in kg) divided by height (in m) squared.

### The spinning sessions

The spinning program lasted 44 min and consisted of continuous cycling, performed alternately seated and standing, at varying target cadence, and at varying target intensity within five target zones. The summarized content of the program including description of the intensity zones is presented in Table 2. The same instructor performed all group and individual sessions. The same music was played in both sessions. Tomahawk IC7 exercise cycles were used (Indoor Cycling Group, Nürnberg, Germany). Each participant used the same cycle for both sessions. Power output and cadence was measured by the exercise cycle. Heart rate was measured with a H9 chest belt (Polar Electro Oy, Kempele, Finland). Data from the exercise cycle and the chest belt were transmitted to a mobile phone installed with Intelligent Cycling software version 8.0.10 (Intelligent Cycling Group ApS, Sorø, Denmark). After each spinning session, enjoyment was determined with a Danish version (6) of the PACES-8 questionnaire (7, 8). The questionnaire consists of eight items and asks participants to rate how they feel at the moment about the physical activity they have been doing. Responses were indicated on a 7-point scale and included choices such as “I find it pleasurable/I find it unpleasurable”. The highest possible sum score, indicating greatest enjoyment, is 56.

**Table 2:** Distribution of the target intensity in the spinning program.

Zone (%)	% of FTP (%)	% of the total session duration (%)	Range of length of bouts (s)
“White”	0–55	10	121–142
“Blue”	56–75	19	17–67
“Green”	76–90	38	12–62
“Yellow”	91–105	21	10–92
“Red”	>106	12	26–46

FTP, functional threshold power output.

### Data analysis

Power output, heart rate, and cadence was sampled at 1 Hz. Average values represent averages across the entire session. Peak values represent the highest measured values in the session.

### Statistical analysis

The results are reported as mean ± standard deviation, unless otherwise stated. The enjoyment data, measured on an interval scale, were analyzed using a Wilcoxon signed-rank test in IBM SPSS 29.0 (IBM Corporation, IBM SPSS Statistics, Armonk, NY, USA). Effect size (Cohen's d) for significant findings was calculated (as dividing the mean difference by the standard deviation of difference) to provide insights into the magnitude of observed difference. The rest of the data were evaluated with a Student's *t*-test in Microsoft Excel 2016 (Microsoft Corporation, Bellevue, WA, USA). Statistical significance was set at *p* < 0.05.

## Results

### BMI

The average BMI for the group of participants fell within the category of overweight. Participants ranged from 20.2 (normal weight) to 33.6 (obese) (9).

### Time of day

There was a statistically nonsignificant difference of 1.4 ± 4.3 h (*p* = 0.097) in the time of day for the two sessions.

### Biomechanical and physiological data

Power output, heart rate, and cadence data from the two spinning sessions are presented in Table 3. There were no statistically significant differences between sessions (*p* > 0.05).

**Table 3:** Biomechanical and physiological responses during the two performed spinning sessions. *n* = 20. Data are presented as mean ± SD across both group and individual spinning sessions, with comparisons on average and peak values for power output, heart rate, and cadence. No statistically significant differences were observed between sessions.

	Power output		Heart rate		Cadence		
	Average (W)	Peak (W)	Average (beats min^−1^)	% of maximal (%)	Peak (beats min^−1^)	Average (rpm)	Peak (rpm)
Session							
Group	167 ± 38	309 ± 66	145 ± 12	85.8 ± 6.8	167 ± 14	70 ± 4	101 ± 10
Individual	169 ± 39	312 ± 94	147 ± 13	86.9 ± 7.4	168 ± 14	69 ± 2	106 ± 19

rpm, revolutions min^−1^.

### Enjoyment

**Figure 1:**
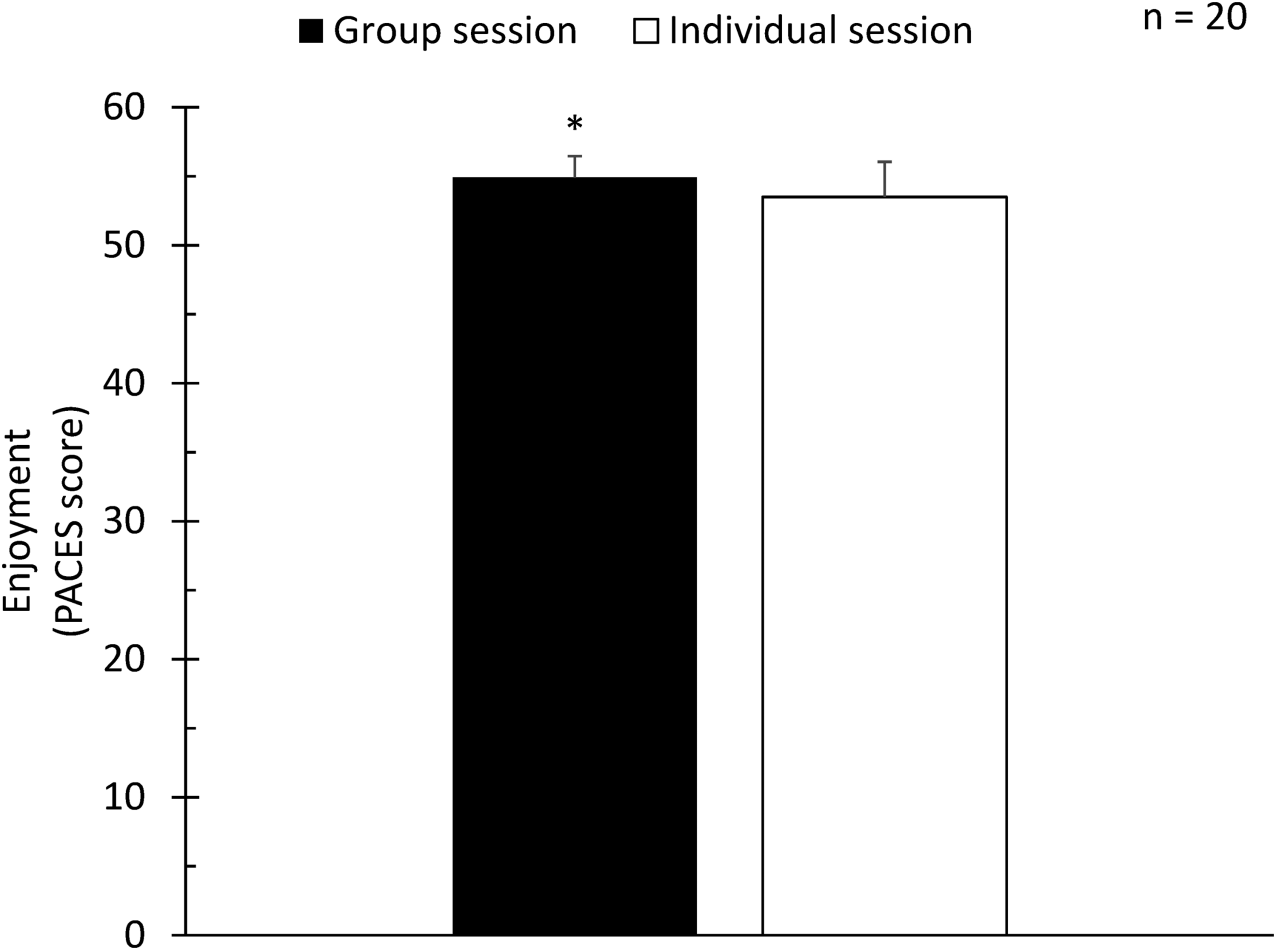
Comparison of self-reported enjoyment scores (PACES-8) between group and individual spinning sessions. Error bars represent standard deviation. An asterisk (*) denotes a statistically significant difference in enjoyment scores (*p* = 0.005) between group and individual sessions. Median and (interquartile range) values for the group and individual sessions were 55.5 (2.0) and 54.5 (2.5), respectively.

**Figure 2:**
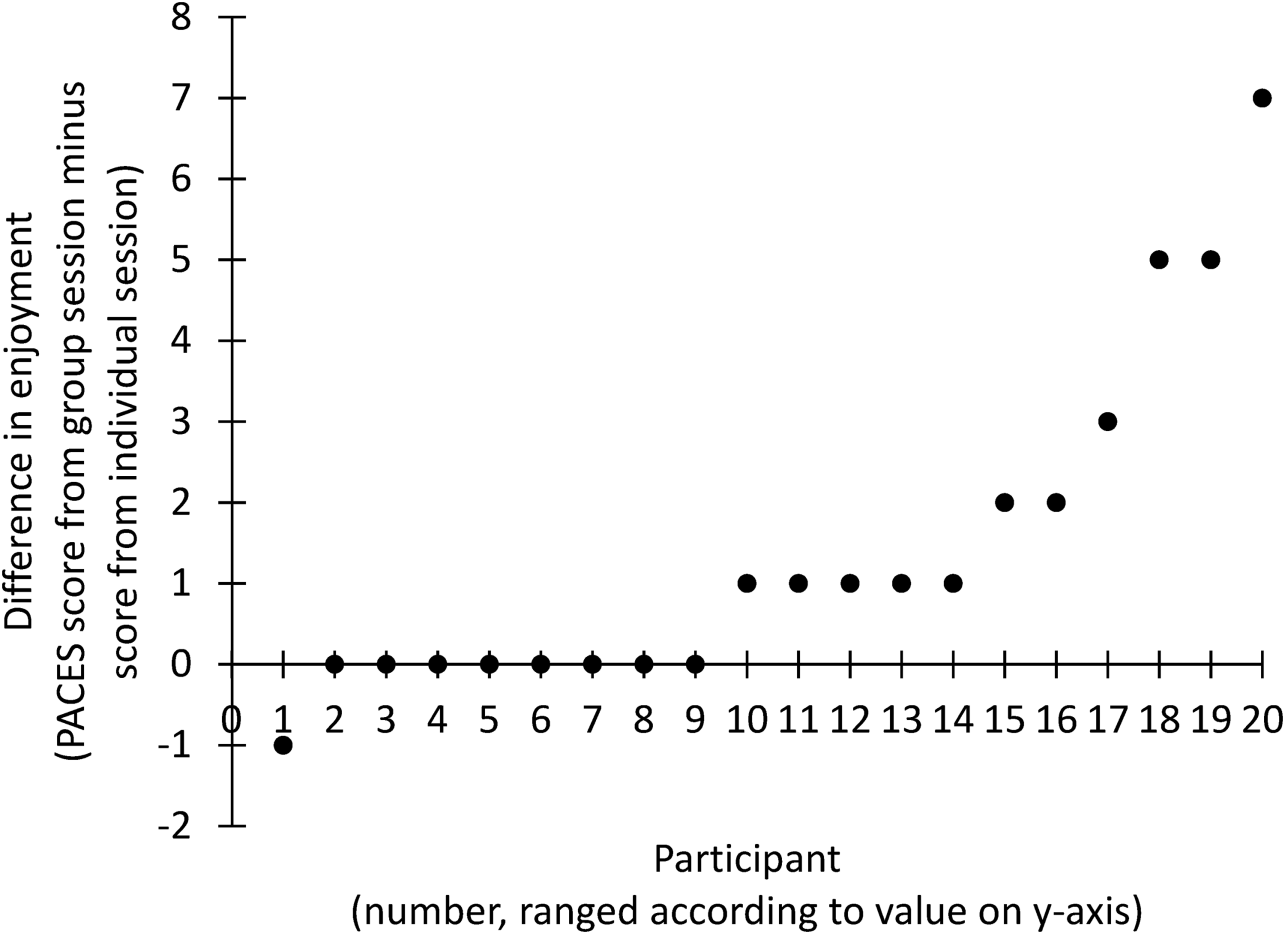
Illustration of individual values of difference in enjoyment between the two performed sessions. A positive value on the *y*-axis represents that the participant's enjoyment was highest in the group session. Of note, all the participants on the *x*-axis are ordered based on the magnitude of the difference.

The magnitude of the difference in PACES-8 score between the two sessions was 1.4 ± 2.1 points (*p* = 0.005) (Figure 1). Effect size was 0.67. Figure 2 illustrates all the individual values of difference between the two sessions. As a maximal value, one participant reported a PACES-8 score that was 7 points higher for the group session as compared to the individual session (Figure 2).

## Discussion

The present study investigated the effect that the group element, in itself, has on enjoyment of a spinning session. This was done by carrying out two instructed spinning sessions that only differed in the way that one of them was performed as a group session while the other was performed individually. It was found that the group element had a statistically significant influence. Although the group element significantly influenced enjoyment, the observed difference was modest, suggesting limited clinical relevance. Additional limitations include the small sample size (*n* = 20) and the specific demography (recreationally active adults). This limits the generalizability of the findings to broader populations or individuals new to spinning. Furthermore, the study's short-term nature, measuring only immediate enjoyment, does not capture potential long-term effects of group dynamics on adherence to physical activity. It is acknowledged that the instructor's behavior could affect the results of a study of the present type. However, a single instructor was used in the present study. The instructor strove to have a similar behavior during all testing. And sessions were performed in random order. Therefore, it is assessed that the instructor's behavior did not explain the observed systematic effect on enjoyment. Lastly, it should be noted that the study relied on self-reported enjoyment that includes potential biases such as recall or social desirability bias.

Other researchers have previously investigated enjoyment of physical activity and exercise and for example focused on the effect of feedback technology (10, 11) and exercise intensity (12, 13).

The measured intensity in the present study corresponds to the boundary between what can be termed vigorous and high (14). Intensity during 50 min of individual spinning has been reported before. Thus, for a group of six women and six men, of on average 30 years and a BMI of 24, the following average values were reported. The men generated 120 W and exercised at 77% of their maximal heart rate. The women generated 73 W and exercised at 82% of their maximal heart rate (15). That intensity is somewhat lower than the intensity recorded in the present study.

In general, the enjoyment of spinning was self-reported to be great by the participants in the present study. A further evaluation of the present results suggests that spinning in a group session results in a statistically significantly greater enjoyment. The greater enjoyment in the group session could for example be due to social facilitation. But further studies have to be performed, to confirm that. Due to the modest size of the observed difference, the clinical difference is assessed to be minor. The results indicate that while group settings may slightly enhance enjoyment, individuals also experience substantial enjoyment in individual sessions. This insight could guide instructors and program developers to offer flexible options, including virtual or solo formats, without significant reductions in participant enjoyment. For those aiming to increase exercise adherence, it might be beneficial to emphasize individual enjoyment in addition to social aspects, especially for populations who may prefer solitary exercise. For the present participants, the group element of the spinning session could be considered being of minor importance for their perception of enjoyment. Of note, this does not exclude that a group element, in itself, of exercise sessions can be a source for considerably enhanced enjoyment in other conditions, for other types of exercise, or for other groups of persons. In relation to the latter, it should be noted that the participants in the present study were characterized by persons who regularly participated in group spinning classes. And they themselves had decided to attend spinning classes. A future study could elucidate the effect of the group element on enjoyment in persons for whom spinning is a new activity. Furthermore, the findings of the present should be tested for reproducibility in further research that at the same time could take into account limitations mentioned above. Also, enjoyment in virtual group settings could be investigated in the future. Finally, it could be considered that for many persons it might be a key to maintenance of physical activity to have an appointment for exercising, which includes that other persons are expecting you to show up. The latter aspect is likely more typically the case for group sessions.

In conclusion, enjoyment was statistically significantly greater during spinning exercise performed in a group session as compared to individually. However, the magnitude of the difference between the two different conditions was modest and likely of minor clinical importance.

## Data Availability

The datasets presented in this study can be found in online repositories (DOI: 10.6084/m9.figshare.26968864). The names of the repository/repositories and accession number(s) can be found in the article/Supplementary Material.
